# Supplementary material for: New classification of superior semicircular canal dehiscence in HRCT
Source: PLoS One. 2022 Jan 20;17(1):e0262758. doi: 10.1371/journal.pone.0262758 (PMC8775191; doi:10.1371/journal.pone.0262758)

PLOS ONE

New classification of superior semicircular canal dehisence in HRCT

--Manuscript Draft--

**Manuscript Number:**	PONE-D-21-25618
**Article Type:**	Research Article
**Full Title:**	New classification of superior semicircular canal dehisence in HRCT
**Short Title:**	SSCD Clasification
**Corresponding Author:**	Stephan Waldeck<br>Bundeswehrzentralkrankenhaus Koblenz<br>Koblenz, GERMANY
**Keywords:**	SSCD types; oVEMP; HRCT; standardized reconstruction algorithm
**Abstract:**	Background and Purpose:<br>The complex anatomy of the temporal bone is difficult to understand and constitutes a challenge in the daily diagnostic routine even for experienced neuroradiologists. In the context of otoneurological (oVEMP) and preoperative diagnostics, the diagnosis of superior semicircular canal dehiscence (SSCD) is of great importance for ENT specialists. The gold standard for this diagnosis is a high-resolution CT (HRCT) of the temporal bone. In order to correctly diagnose SSCD, special oblique reconstructions are necessary in addition to standard (axial, coronal, sagittal) reconstructions. We evaluated the frequency of diagnosis and its location in HRCT in correlation with otoneurological examination. From this analysis, we present an SSCD classification.<br><br>Study design and setting:<br>We evaluated 1370 temporal bone scans of patients with residual hearing and verified 343 superior semicircular canal dehiscence (SSCD). We conducted a subgroup analysis of these 343 HRCT scans displaying a SSCD and used them as a basis to create a classification.<br><br>Results:<br>Three location types of SSCD were identified. These were anterior type 1, superior type 2 and posterior type 3. Type 2 were significantly more frequent in both sexes. SSCD at this location can be overlooked if diagnosis is performed only in the standard axial plane, since it can only be visualized by means of double oblique reconstruction. We present a standardized reconstruction algorithm.<br><br>Conclusion:<br>In total, three types of SSCD with differing incidences can be extrapolated from the locations. Superior type 2 is the most frequent one. Both sexes are affected with roughly equal incidence. The use of standardized double oblique reconstruction algorithm ensures that all three types are diagnosed in the HRCT.
**Order of Authors:**	Stephan Waldeck
	Heinrich Lanfermann
	Christian von Falck
	Matthias F Froelich
	René Chapot
	Marc Brockmann
	Daniel Overhoff
**Additional Information:**	
**Question**	**Response**
**Financial Disclosure**	The author(s) received no specific funding for this work.

Powered by Editorial Manager® and ProduXion Manager® from Aries Systems Corporation

Enter a financial disclosure statement that describes the sources of funding for the work included in this submission. Review the submission guidelines for detailed requirements. View published research articles from *PLOS ONE* for specific examples.<br><br>This statement is required for submission and **will appear in the published article** if the submission is accepted. Please make sure it is accurate.<br><br>**Unfunded studies**<br>Enter: *The author(s) received no specific funding for this work.*<br><br>**Funded studies**<br>Enter a statement with the following details:<br>• Initials of the authors who received each award<br>• Grant numbers awarded to each author<br>• The full name of each funder<br>• URL of each funder website<br>• Did the sponsors or funders play any role in the study design, data collection and analysis, decision to publish, or preparation of the manuscript?<br>• **NO** - Include this sentence at the end of your statement: *The funders had no role in study design, data collection and analysis, decision to publish, or preparation of the manuscript.*<br>• **YES** - Specify the role(s) played.<br><br>* typeset	
**Competing Interests**<br><br>Use the instructions below to enter a competing interest statement for this submission. On behalf of all authors, disclose any competing interests that could be perceived to bias this work—acknowledging all financial support and any other relevant financial or non-financial competing interests.<br><br>This statement is required for submission and **will appear in the published article** if the submission is accepted. Please make	The authors have declared that no competing interests exist.

sure it is accurate and that any funding sources listed in your Funding Information later in the submission form are also declared in your Financial Disclosure statement.<br><br>View published research articles from *PLOS ONE* for specific examples.<br><br>**NO authors have competing interests**<br><br>Enter: *The authors have declared that no competing interests exist.*<br><br>**Authors with competing interests**<br><br>Enter competing interest details beginning with this statement:<br><br>*I have read the journal's policy and the authors of this manuscript have the following competing interests: [insert competing interests here]*<br><br>* typeset	
**Ethics Statement**<br><br>Enter an ethics statement for this submission. This statement is required if the study involved:<br><br>• Human participants<br>• Human specimens or tissue<br>• Vertebrate animals or cephalopods<br>• Vertebrate embryos or tissues<br>• Field research<br><br>Write "N/A" if the submission does not require an ethics statement.<br><br>General guidance is provided below. Consult the submission guidelines for detailed instructions. **Make sure that all information entered here is included in the Methods section of the manuscript.**	Local ethics committee (“Ethikkommission Landesärztekammer Rheinland-Pfalz”) approved this retrospective study (Nr: 2018-13750).<br><br>For this retrospective study only pseudonymized medical records were used.<br>Ethics committee waived the requirement for informed consent due to the retrospective nature of this study.<br><br>, please ensure that you have discussed whether all data were fully anonymized before you accessed them and/or whether the IRB or ethics committee waived the requirement for informed consent.

Powered by Editorial Manager® and ProduXion Manager® from Aries Systems Corporation

**Format for specific study types**<br><br>**Human Subject Research (involving human participants and/or tissue)**<br>• Give the name of the institutional review board or ethics committee that approved the study<br>• Include the approval number and/or a statement indicating approval of this research<br>• Indicate the form of consent obtained (written/oral) or the reason that consent was not obtained (e.g. the data were analyzed anonymously)<br><br>**Animal Research (involving vertebrate animals, embryos or tissues)**<br>• Provide the name of the Institutional Animal Care and Use Committee (IACUC) or other relevant ethics board that reviewed the study protocol, and indicate whether they approved this research or granted a formal waiver of ethical approval<br>• Include an approval number if one was obtained<br>• If the study involved *non-human primates*, add *additional details* about animal welfare and steps taken to ameliorate suffering<br>• If anesthesia, euthanasia, or any kind of animal sacrifice is part of the study, include briefly which substances and/or methods were applied<br><br>**Field Research**<br><br>Include the following details if this study involves the collection of plant, animal, or other materials from a natural setting:<br>• Field permit number<br>• Name of the institution or relevant body that granted permission	
**Data Availability**<br><br>Authors are required to make all data underlying the findings described fully available, without restriction, and from the time of publication. PLOS allows rare exceptions to address legal and ethical concerns. See the PLOS Data Policy and FAQ for detailed information.	Yes - all data are fully available without restriction

Powered by Editorial Manager® and ProduXion Manager® from Aries Systems Corporation

A Data Availability Statement describing where the data can be found is required at submission. Your answers to this question constitute the Data Availability Statement and **will be published in the article**, if accepted.<br><br>**Important:** Stating 'data available on request from the author' is not sufficient. If your data are only available upon request, select 'No' for the first question and explain your exceptional situation in the text box.<br><br>Do the authors confirm that all data underlying the findings described in their manuscript are fully available without restriction?	
**Describe where the data may be found in full sentences. If you are copying our sample text, replace any instances of XXX with the appropriate details.**<br><br>• If the data are **held or will be held in a public repository**, include URLs, accession numbers or DOIs. If this information will only be available after acceptance, indicate this by ticking the box below. For example: *All XXX files are available from the XXX database (accession number(s) XXX, XXX.).*<br>• If the data are all contained **within the manuscript and/or Supporting Information files**, enter the following: *All relevant data are within the manuscript and its Supporting Information files.*<br>• If neither of these applies but you are able to provide **details of access elsewhere**, with or without limitations, please do so. For example:<br><br>*Data cannot be shared publicly because of [XXX]. Data are available from the XXX Institutional Data Access / Ethics Committee (contact via XXX) for researchers who meet the criteria for access to confidential data.*<br><br>*The data underlying the results presented in the study are available from (include the name of the third party*	Data will not be held in a public repository due to the restrictions of the ehtics committee.

and contact information or URL).<br>• This text is appropriate if the data are owned by a third party and authors do not have permission to share the data.<br><br>* typeset	
Additional data availability information:	

Powered by Editorial Manager® and ProduXion Manager® from Aries Systems Corporation

Manuscript
Click here to access/download;Manuscript;SSCD_final_sub.docx

New Classification of superior semicircular canal dehiscence in HRCT

Stephan Waldeck[1,6], Heinrich Lanfermann[2], Christian von Falck[3], Matthias F Froelich[4] René Chapot[5], Marc Brockmann[6], Daniel Overhoff[1,4]

1. Department of Diagnostic and Interventional Radiology and Neuroradiology, Bundeswehr Central Hospital Koblenz, Rübenacher Straße 170, 56072 Koblenz, Germany
2. Institute of Neuroradiology, Hannover Medical School, Carl-Neuberg-Straße 1, 30625 Hanover, Germany
3. Institute of Diagnostic and Interventional Radiology, Hannover Medical School, Carl-Neuberg-Straße 1, 30625 Hanover, Germany
4. Department of Radiology and Nuclear Medicine, University Medical Centre Mannheim, Medical Faculty Mannheim, Heidelberg University, Theodor-Kutzer-Ufer 1-3, 68167 Mannheim, Germany
5. Department of Neuroradiology, Alfried Krupp Krankenhaus, Alfried-Krupp-Strasse 21, 45131 Essen, Germany
6. Institute of Neuroradiology, University Medical Centre Johannes Gutenberg University Mainz, Langenbeckstraße 1, 55131 Mainz, Germany

Address for Correspondence:

Stephan Waldeck, MD

Rübenacherstrassse 170, 56072 Koblenz, Germany

Phone: (+49) (+261) – 281 – 28000

Email: dr.waldeck@icloud.com

Fax: (+49) (+261) – 281 – 28009

Disclosures: No financial conflict of interest

Short Title: SSCD Clasification

Abstract

Background and Purpose:

The complex anatomy of the temporal bone is difficult to understand and constitutes a challenge in the daily diagnostic routine even for experienced neuroradiologists. In the context of otoneurological (oVEMP) and preoperative diagnostics, the diagnosis of superior semicircular canal dehiscence (SSCD) is of great importance for ENT specialists. The gold standard for this diagnosis is a high-resolution CT (HRCT) of the temporal bone. In order to correctly diagnose SSCD, special oblique reconstructions are necessary in addition to standard (axial, coronal, sagittal) reconstructions. We evaluated the frequency of diagnosis and its location in HRCT in correlation with otoneurological examination. From this analysis, we present an SSCD classification.

Study design and setting:

We evaluated 1370 temporal bone scans of patients with residual hearing and verified 343 superior semicircular canal dehiscence (SSCD). We conducted a subgroup analysis of these 343 HRCT scans displaying a SSCD and used them as a basis to create a classification.

Results:

Three location types of SSCD were identified. These were anterior type 1, superior type 2 and posterior type 3. Type 2 were significantly more frequent in both sexes. SSCD at this location can be overlooked if diagnosis is performed only in the standard axial plane, since it can only be visualized by means of double oblique reconstruction. We present a standardized reconstruction algorithm.

Conclusion:

In total, three types of SSCD with differing incidences can be extrapolated from the locations. Superior type 2 is the most frequent one. Both sexes are affected with roughly equal incidence. The use of standardized double oblique reconstruction algorithm ensures that all three types are diagnosed in the HRCT.

Keywords: SSCD types, oVEMP, HRCT, standardized reconstruction algorithm

Abbreviations

HRCT	high resolution computed tomography
SSC	superior semicircular canal
SSCD	superior semicircular canal dehiscence
oVEMP	ocular vestibular evoked myogenic potential

Introduction:

The clinical picture of superior semicircular canal dehiscence (SSCD) was described for the first time by Minor et al. in 1998 (1). Patients reported vestibular (vertigo, nystagmus, Tullio phenomenon, Hennebert sign (2)) and acoustic signs and symptoms (conductive or sensorineural hearing loss, autophony, tinnitus).

These problems typically occur in nearly all age groups. The average age for the onset of this illness is during middle age (38–61 years) (2-5). In radiological studies the prevalence has been stated to be between 2.1% and 20.4% (6-9).

Pathophysiologically, the signs and symptoms can be explained by the development of a “third window” caused by an absence of the bone overlying the superior semicircular canal (SSC). This results in a connection between the inner ear and the middle cranial fossa, which is described as a “third window” in addition to the oval and round windows. Pressure changes can trigger endolymph movement in the superior semicircular canal, which can ultimately lead to an excitation of the cupula and cause the described signs and symptoms (10,11).

The etiology of SSCD is yet to be explained conclusively. Both inflammatory and traumatic causes are being discussed. In the case of trauma, a tearing of the thin superior semicircular canal membrane occurs and the onset of symptoms is instantaneous (12-14). Figure 1 shows the mechanism that can lead to the symptoms (Figure 1).

High-resolution computed tomography (HRCT) is an essential part of diagnosing SSCD since it offers a high degree of sensitivity (17). The positive predictive value is given as 93% for a slice thickness of 0.5 mm (18,19). Sensitivity decreases dramatically for thicker slices. It is important to understand that SSCD location can differ. With regard to its location in the superior semicircular canal, dehiscence can be anterior, superior or posterior. In order to make sure that all location types are detected, special reconstructions must be generated from the submillimeter slices.

This retrospective multicenter study analyzed 1370 HRCT scans of the temporal bone and verified 343 SSCDs. Their distribution was grouped into three types. The evaluation also included an examination of the incidence of the location types in relation to sex and age.

Materials and Methods:

Study design

A retrospective multicenter study of high-resolution CT scan from patients with vestibular and/or acoustic symptoms. Local ethics committee ("Ethikkommission Landesärztekammer Rheinland-Pfalz") approved this retrospective study (Nr: 2018-13750). For this retrospective study only pseudonymized medical records were used. Ethics committee waived the requirement for informed consent due to the retrospective nature of this study.

Patient cohort:

We analyzed 1370 HRCT scans of the temporal bone from ENT patients (from May 2008 to March 2018) for the presence of SSCDs. For all diagnosed SSCD we performed a subgroup analysis. These were subdivided by gender und grouped into four age groups (A: 6–24, B:25–49, C:50–74, D:75–95 years).

Data Collection:

The CT examinations of the temporal bone obtained from the different institutes and departments were conducted based on the following parameters:

Koblenz: Siemens Somatom Force 384-slice scanner; 120 kV; 140 mAs; 0.80 pitch.

Toshiba Vision Edition 320; 120 kV; 200 mAs; 16 cm volume scan

Mainz: Toshiba Aquilon 32-slice scanner; 120 kV; 200 mAs; 0.66 pitch

Hanover: GE LightSpeed 16; 100 kV; 80 mAs; 1.38 pitch

A slice thickness of 0.6 mm was used for multiplanar reconstruction. All data were analyzed with Siemens Syngo Via software (version VB10).

Standardized reconstruction scheme:

In order to detect all SSCDs, the image analysis followed a standardized reconstruction scheme on the basis of the MPRs. The image reconstruction and the subgroup analysis of the SSCD localization based on it were carried out in 4 steps:

1: The axial data set is used to generate a reconstruction orthogonal to the superior semi-circular canal with a slice thickness of 0.6 mm. (Figure 2a).

2: This reconstruction is used to generate another oblique reformation parallel to the SSC with a slice thickness of 0.6 mm. (Figure 2a).

3: The result of the 2 reformations[1] is used to create an MIP image (1mm) to render the entire SSC visible in one image. (Figure 2b).

4: The locations of dehiscence in the superior semicircular canal (SSC) were grouped into three types (Figure 2c).

Statistical analysis

The statistical calculations were performed using SPSS (IBM SPSS Statistics, version 20.0 for Macintosh; SPSS, Inc., Chicago, IL, USA).

All continuous variables are expressed as arithmetic mean ± standard deviation (SD). A significance level of 5% was used. Normal distribution was assumed due to central limit theorem. For continuous data Student`s t- Test was performed for independent samples to analyze the means. To analyze ordinal data Chi- square test was used.

Results

We analyzed 1370 high resolution CT scans of the temporal bone from ENT patients (681 female (49,7%) versus 689 male (50,3%) for the presence of SSCDs und verified 343 diagnoses out of 280 patients (prevalence of 20.42%). Patients with SSCD were on average 6 years older than those without SSCD (mean 46.22 years versus 52.73 years (SSCD) p<0.001).

63 patients had a diagnosis of bilateral SSCD, 116 unilateral left, 101 unilateral right; unilateral/bilateral ratio of 3.44:1. Table 1 summarizes all the results of the gender in relation to the frequency of SSCD types and its locations.

We were able to visualize all SSCD types with the standardized reconstruction scheme (Figure 3).

The total of 280 patients with SSCD (123 female and 157 male; p=0.030) were grouped into four age groups (A:6–24, B:25–49, C:50–74, D:75–95 years).

Of a total of 343 SSCD, 99 (28,86 %) displayed dehiscence of an anterior location type (type 1), 133 (38,78 %) displayed the dominant superior location type (type 2) and 111 (32,36 %) displayed the posterior location type (type 3). The different Types are shown in Figure 4.

SSCD type 2 is significantly more common in males (p=0.048) and significantly more common in age group C (p=0.012) and D (p=-0.004).

SSCD type 1 and SSCD type 3 are not significantly more common in all age groups. Overall, no significant difference was evident between the sexes in terms of incidence.

The age-correlated increase of SSCDs lead to a significant change in the location of dehiscence. There was a dominance of the superior location of dehiscence corresponding to type 2 in 3 of the 4 age groups (group 2: 25-49 y; group 3: 50-74 y; group 4: 75-95 y). Across all age groups, SSCD type 2 is significantly more common than type 3 and this than type 1 (type 2 > type3 > type1).

Table 2 summarizes all the results of the different age groups in relation to the frequency of SSCD types.

Table 1: Study Population

Population n= 1370	SSCD		
**sex**	**male**	***female***	
	157 (22,8%)	*123 (18,2%)*	
**side**	***right***	***left***	**both**
	101 (7,4%)	116 (8,5%)	63 (4,6%)
**type**	**1**	**2**	**3**
	99 (7,2%))	133 (9,7%)	111 (8,1%)

Table 1. sex relative values to the sex; side, type relative values to total population

Table 2: Differentiation of SSCD types by age cohorts.

	0-24 yo	25-49 yo	50-74 yo	>75 yo	all ages
**SSCD Type 1**	**19 (2.7%)**	***26 (3.5%)***	**27*(3.3%)***	**27*(5.0%)***	**99(7.2%)**
**SSCD Type 2**	**26 (3.7%)**	***32 (4.3%)***	**39*(5.2%)***	**36*(6.7%)***	**133*(9.7%)***
**SSCD Type 3**	**20*(2.8%)***	***25(3.4%)***	**36*(5.0%)***	**30*(5.6%)***	**111*(8.1%)***
**SSCD Type1-3**	**65*(9.2%)***	***83(11.2%)***	**102*(13.5%)***	**93*(17.2%)***	**343*(25.0%)***

Table 2. Relative values in percent in parentheses. The relative values of the age cohort columns refer to the respective age cohort. The relative values of the "all ages" columns refer to the total study population.

Discussion

Superior Semicircular Canal Dehiscence (SSCD) is still a relatively new pathologic condition that requires a high suspicion for its correct diagnosis. While different surgical approaches and techniques have been published in the literature, the exact location of the SSCD is of high importance. To the best of our knowledge the presented analysis includes a considerably larger number of cases than other published radiological studies before (8). We are the first to analyze the location of superior semicircular canal dehiscence based on a fixed reconstruction scheme. CT is still said to result in an overdiagnosis of SSCD, especially for slice thicknesses of ≥ 1 mm (7). Nevertheless, it is an integral part of equipment-based diagnostics of SSCD in case of clinical suspicion, especially if it was done in high resolution.

In our study all multiplanar reconstructions were evaluated based on sub-millimeter slices and complemented by standardized double-oblique reconstruction (19-23). Our patient group displayed a relative dominance of the type 2 location regardless of sex and age. This SSCD type 2 can be overlooked in standard axial reconstructions because it is in the superior semicircular canal apex. We were able to reliably visualize SSCD location type 2 in all cases using the reconstruction algorithm presented here.

343 SSCD data sets from 280 patients were analyzed for our subgroup analysis. The data originated from an analysis of 1370 petrous temporal bone HRCTs. At a prevalence of up to 20.42%, SSCD is a new but not rare diagnosis in patients with residual hearing. The results largely correspond to those of other radiological studies (Nadgir et al.: 7.9% (n = 304) (24); Stimmer et al.: 12.6% (n = 350) (8); Williamson et al.: 13.5% (n= 223) (7); Ceylan et al.: 17.5% (n = 108) (9)).

It is important to know about and correctly diagnose SSCD. Our reconstruction scheme notably reduces the risk of overdiagnosis when using CT. The risk of overdiagnosis is especially large if slice thicknesses of ≥ 1 mm are used. Belden et al. demonstrated the importance of selecting a slice thickness of < 1 mm in diagnosing SSCD when they disproved in all 36 cases an initial suspicion of SSCD formed after acquisition based on 1 mm slice thickness by re-examining the cases with 0.5 mm slices (18). We therefore recommend the use of a scan protocol with a slice thickness of ≤ 0.6 mm.

The present retrospective analysis showed overall age groups no significant difference in the relative incidence of SSCD for women and men. In the subgroup analysis SSCD type 2 is significantly more common in males and significantly more common in age

group C and D. These results are in line with the observations made by Crovetto et al. (23), da Cunha Ferreira et al. (25) and Martin et al. (26).

Study limitations:

Our retrospective study focused on the incidental findings of SSCD in the HRCT as primary outcome. A multiparametric analysis with respect to clinical parameters such as vestibular symptoms or in the correlation of new high frequency ocular vestibular evoked myogenic potential (oVEMP n10 response) (27) for the study cohort were not the primary target of this study.

Statistically reliable investigations about the different SSCD types in comparison with the clinical parameters remain to be conducted in in a prospective study.

Conclusion

From the neuroradiological point of view, the relatively new SSCD diagnosis with its prevalence of up to 20.42% among individuals with residual hearing is not a rare one. This makes it all the more important for the radiologist to obtain certainty by confirming the diagnosis by means of HRCT. Our reconstruction scheme makes a reliable visualization and clear classification of SSCD possible which can be of great importance for the operational approach.

References:

1. Minor LB, Solomon D, Zinreich JS, Zee DS. Sound- and/or pressure-induced vertigo due to bone dehiscence of the superior semicircular canal. Archives of otolaryngology--head & neck surgery. 1998;124(3):249-58. Epub 1998/04/03.
2. Chi FL, Ren DD, Dai CF. Variety of audiologic manifestations in patients with superior semicircular canal dehiscence. Otology & neurotology : official publication of the American Otological Society, American Neurotology Society [and] European Academy of Otology and Neurotology. 2010;31(1):2-10. Epub 2010/01/06.
3. Strupp M, Eggert T, Straube A, Jager L, Querner V, Brandt T. ["Inner perilymph fistula" of the anterior semicircular canal. A new disease picture with recurrent attacks of vertigo]. Der Nervenarzt. 2000;71(2):138-42. Epub 2000/03/07. "Innere Perilymphfistel" des anterioren Bogengangs. Ein neues Krankheitsbild mit rezidivierenden Schwindelattacken.
4. Minor LB, Carey JP, Cremer PD, Lustig LR, Streubel S-O. Dehiscence of bone overlying the superior canal as a cause of apparent conductive hearing loss. Otology & Neurotology. 2003;24(2):270-8.
5. Minor LB. Clinical manifestations of superior semicircular canal dehiscence. Laryngoscope. 2005;115(10):1717-27. Epub 2005/10/14.
6. Loke SC, Goh JP. Incidence of semicircular canal dehiscence in Singapore. The British journal of radiology. 2009;82(977):371-3. Epub 2008/12/23.
7. Williamson RA, Vrabec JT, Coker NJ, Sandlin M. Coronal computed tomography prevalence of superior semicircular canal dehiscence. Otolaryngology--head and neck surgery : official journal of American Academy of Otolaryngology-Head and Neck Surgery. 2003;129(5):481-9. Epub 2003/11/05.
8. Stimmer H, Hamann KF, Zeiter S, Naumann A, Rummeny EJ. Semicircular canal dehiscence in HR multislice computed tomography: distribution, frequency, and clinical relevance. European archives of oto-rhino-laryngology : official journal of the European Federation of Oto-Rhino-Laryngological Societies (EUFOS) : affiliated with the German Society for Oto-Rhino-Laryngology - Head and Neck Surgery. 2012;269(2):475-80. Epub 2011/07/09.
9. Ceylan N, Bayraktaroglu S, Alper H, Savas R, Bilgen C, Kirazli T, et al. CT imaging of superior semicircular canal dehiscence: added value of reformatted images. Acta oto-laryngologica. 2010;130(9):996-1001. Epub 2010/03/09.

10. Banerjee A, Whyte A, Atlas MD. Superior canal dehiscence: review of a new condition. Clin Otolaryngol. 2005;30(1):9-15.
11. Tsunoda A, Terasaki O. Dehiscence of the bony roof of the superior semicircular canal in the middle cranial fossa. The Journal of laryngology and otology. 2002;116(7):514-8. Epub 2002/09/20.
12. Mehlenbacher A, Capehart B, Bass D, Burke JR. Sound induced vertigo: superior canal dehiscence resulting from blast exposure. Archives of physical medicine and rehabilitation. 2012;93(4):723-4. Epub 2012/01/06.
13. Adams ME, Levine SC. The first new otologic disorder in a century: superior canal dehiscence syndrome. Minnesota medicine. 2011;94(11):29-32. Epub 2012/03/15.
14. Ogutha J, Page NC, Hullar TE. Postpartum vertigo and superior semicircular canal dehiscence syndrome. Obstetrics and gynecology. 2009;114(2 Pt 2):434-6. Epub 2009/07/23.
15. Phillips DJ, Souter MA, Vitkovic J, Briggs RJ. Diagnosis and outcomes of middle cranial fossa repair for patients with superior semicircular canal dehiscence syndrome. Journal of clinical neuroscience : official journal of the Neurosurgical Society of Australasia. 2010;17(3):339-41. Epub 2010/01/16.
16. Belden CJ, Weg N, Minor LB, Zinreich SJ. CT evaluation of bone dehiscence of the superior semicircular canal as a cause of sound- and/or pressure-induced vertigo. Radiology. 2003;226(2):337-43. Epub 2003/02/04.
17. Crane BT, Minor LB, Carey JP. Three-dimensional computed tomography of superior canal dehiscence syndrome. Otology & neurotology : official publication of the American Otological Society, American Neurotology Society [and] European Academy of Otology and Neurotology. 2008;29(5):699-705. Epub 2008/06/04.
18. Brantberg K, Verrecchia L. Testing vestibular-evoked myogenic potentials with 90-dB clicks is effective in the diagnosis of superior canal dehiscence syndrome. Audiology and Neurotology. 2008;14(1):54-8.
19. Brantberg K, Bergenius J, Tribukait A. Vestibular-evoked myogenic potentials in patients with dehiscence of the superior semicircular canal. Acta oto-laryngologica. 1999;119(6):633-40.
20. Zhou G, Gopen Q, Poe DS. Clinical and diagnostic characterization of canal dehiscence syndrome: a great otologic mimicker. Otology & neurotology : official

publication of the American Otological Society, American Neurotology Society [and] European Academy of Otology and Neurotology. 2007;28(7):920-6. Epub 2007/10/24.

21. Cloutier JF, Belair M, Saliba I. Superior semicircular canal dehiscence: positive predictive value of high-resolution CT scanning. European archives of oto-rhino-laryngology : official journal of the European Federation of Oto-Rhino-Laryngological Societies (EUFOS) : affiliated with the German Society for Oto-Rhino-Laryngology - Head and Neck Surgery. 2008;265(12):1455-60. Epub 2008/04/17.
22. Nadgir RN, Ozonoff A, Devaiah AK, Halderman AA, Sakai O. Superior semicircular canal dehiscence: congenital or acquired condition? AJNR American journal of neuroradiology. 2011;32(5):947-9. Epub 2011/03/12.
23. Crovetto M, Whyte J, Rodriguez OM, Lecumberri I, Martinez C, Elexpuru J. Anatomo-radiological study of the Superior Semicircular Canal Dehiscence Radiological considerations of Superior and Posterior Semicircular Canals. European journal of radiology. 2010;76(2):167-72. Epub 2009/06/23.
24. Minor LB, Cremer PD, Carey JP, Della Santina CC, Streubel SO, Weg N. Symptoms and signs in superior canal dehiscence syndrome. Annals of the New York Academy of Sciences. 2001;942:259-73. Epub 2001/11/17.
25. Crovetto MA, Whyte J, Rodriguez OM, Lecumberri I, Martinez C, Fernandez C, et al. Influence of aging and menopause in the origin of the superior semicircular canal dehiscence. Otology & neurotology : official publication of the American Otological Society, American Neurotology Society [and] European Academy of Otology and Neurotology. 2012;33(4):681-4. Epub 2012/05/10.
26. Joakimsen O, Bønaa KH, Stensland-Bugge E, Jacobsen BK. Population-based study of age at menopause and ultrasound assessed carotid atherosclerosis: The Tromsø Study. Journal of clinical epidemiology. 2000;53(5):525-30.
27. Lin K, Lahey R, Beckley R, Bojrab D 2nd, Wilkerson B, Johnson E, Babu S, Hong RS. Validating the Utility of High Frequency Ocular Vestibular Evoked Myogenic Potential Testing in the Diagnosis of Superior Semicircular Canal Dehiscence. Otol Neurotol. 2019 Dec;40(10):1353-1358. doi:10.1097/MAO.0000000000002388. PMID: 31725592.

Figures:

Figure 1: SSCD symptoms scheme:

Figure 1. The Arrows show the mechanism that can lead to the SSCD symptoms, with both intracranial pressure increases and otoacoustic stimuli acting as possible triggers.

Figure 2a-b-c: Standardized Reconstruction algorithm and SSCD Classification: Type 1-3 :

Figure 2 uses a model to illustrate the reconstruction scheme.

Figure 3: HR CT Reconstruction:

Figure 3 illustrates the practical implementation with a temporal bone preparation.

Figure 4: SSCD location types:

Figure 4 shows the three different SSCD Types in high resolution CT.

Figure1

Click here to access/download;Figure;Figure 1.png

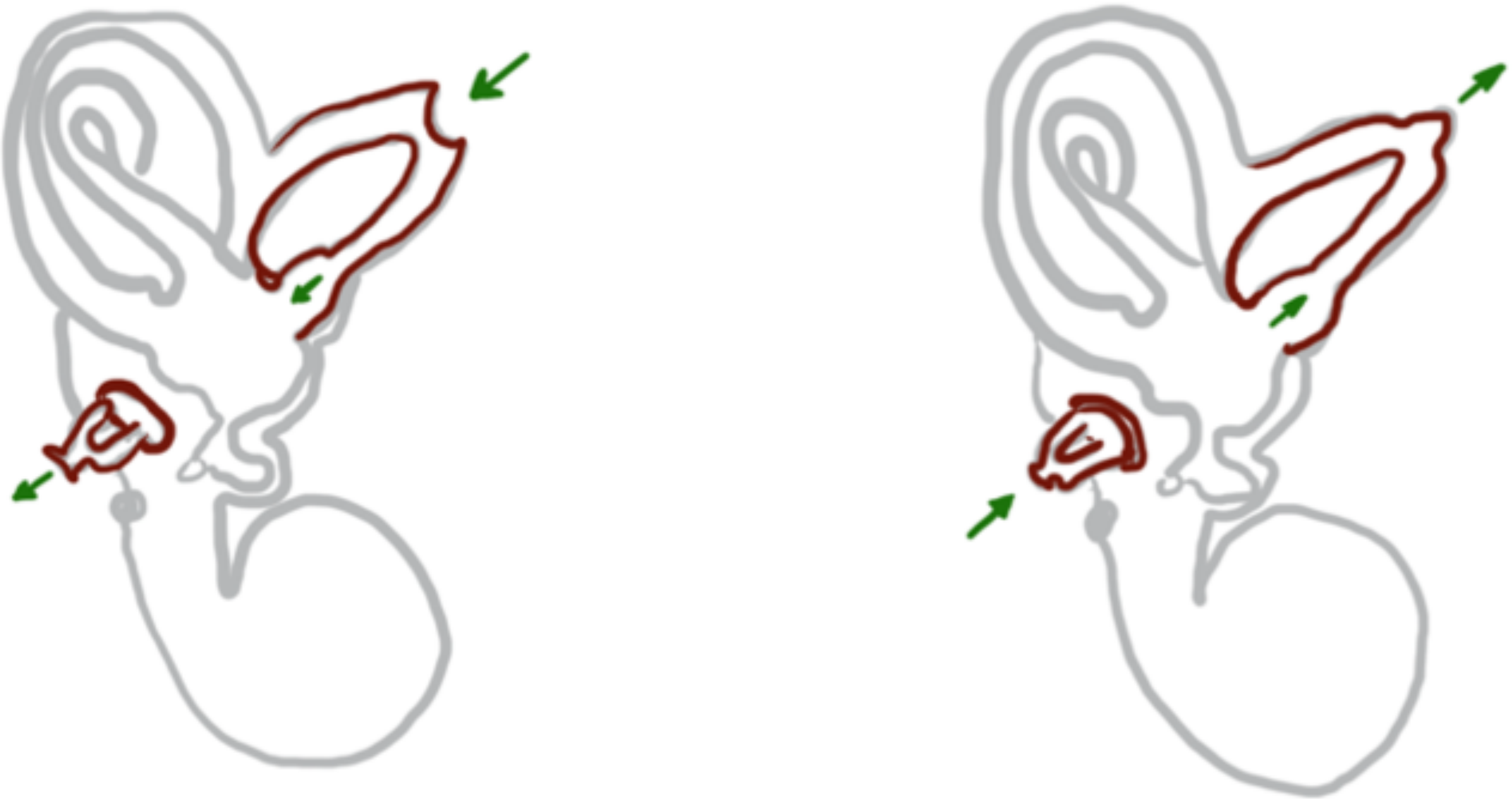

Figure2a

Click here to access/download;Figure;Figure2a.png

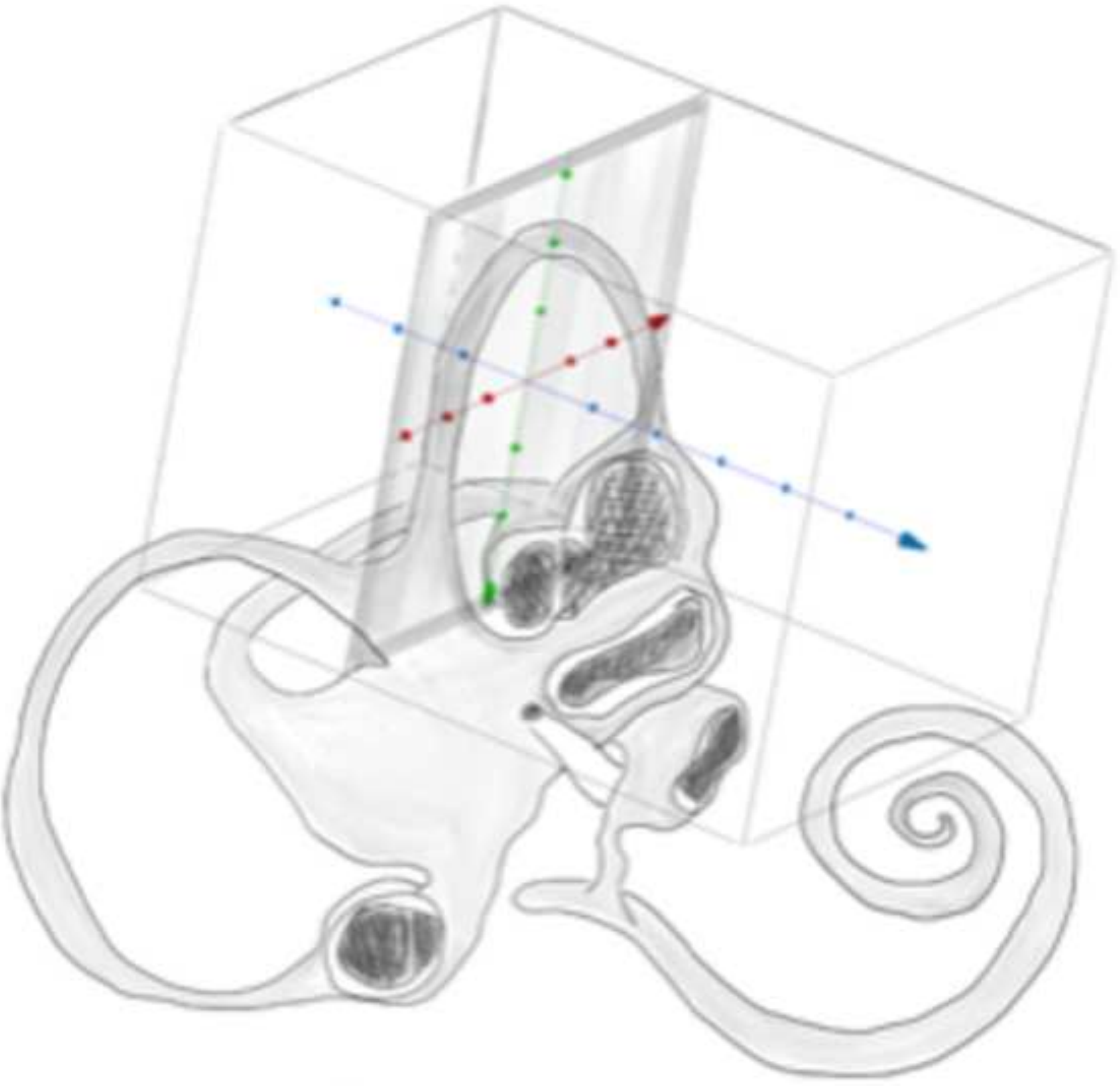

Figure2b

Click here to access/download;Figure;Figure2b.png

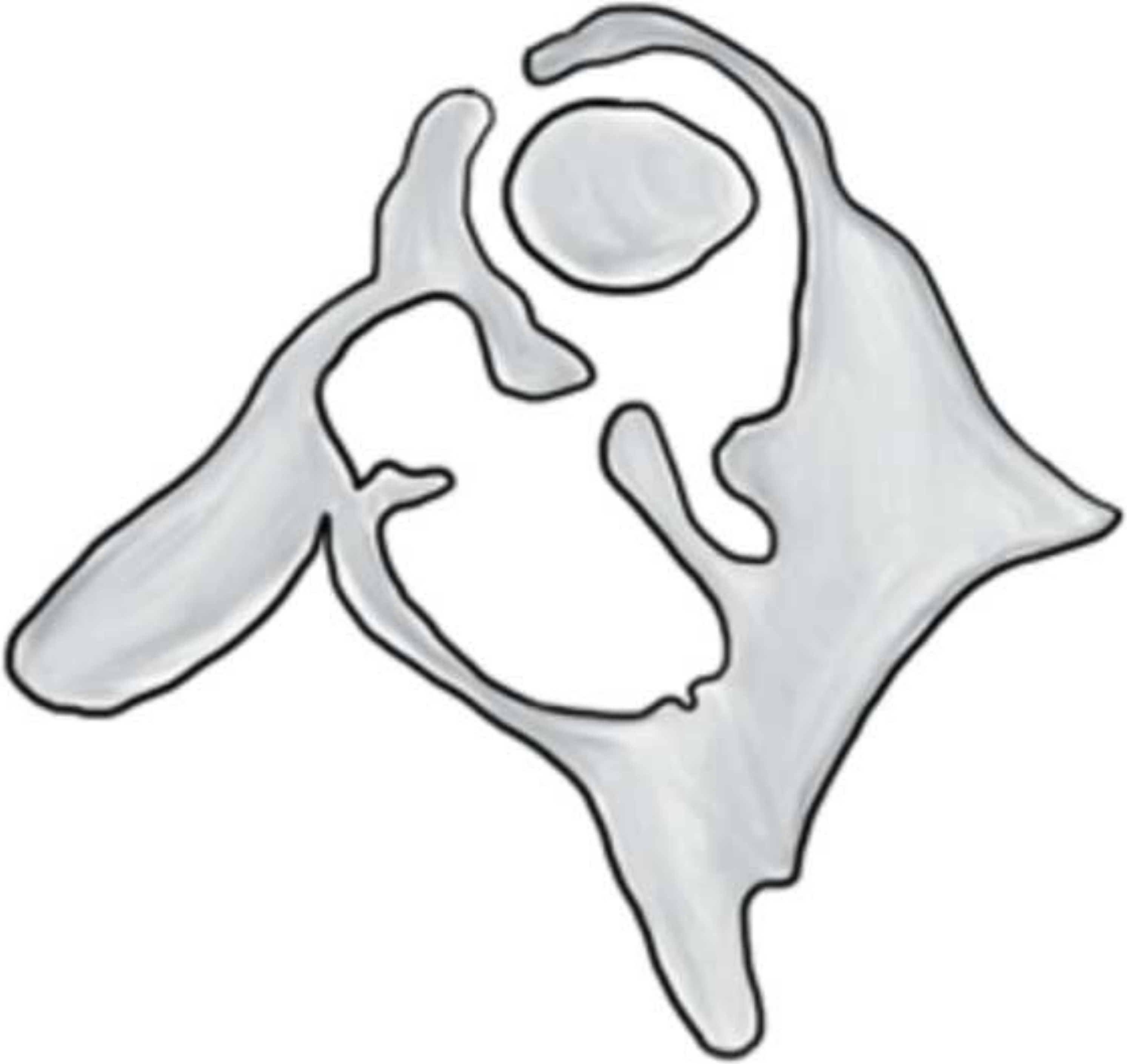

Figure2c

Click here to access/download;Figure;Figure2c.png

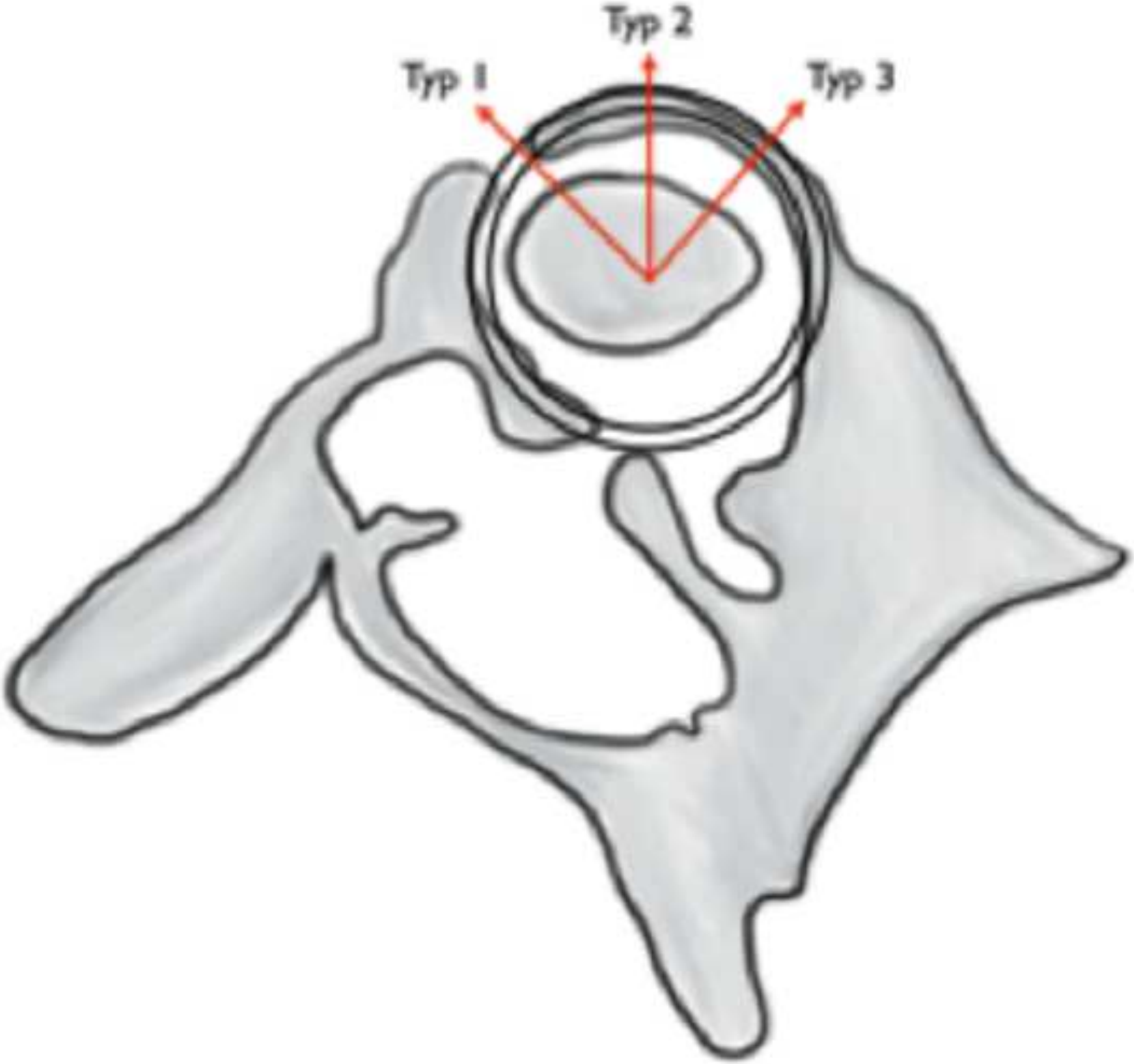

Figure3 Click here to access/download;Figure;Figure3.png

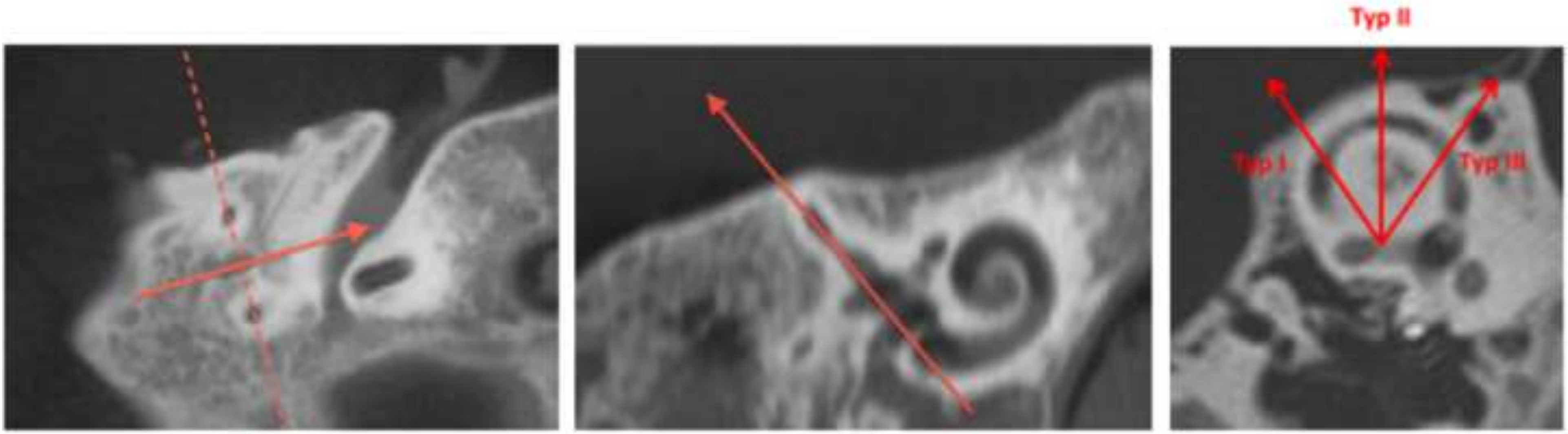

 Click here to access/download;Figure;Figure4.jpg

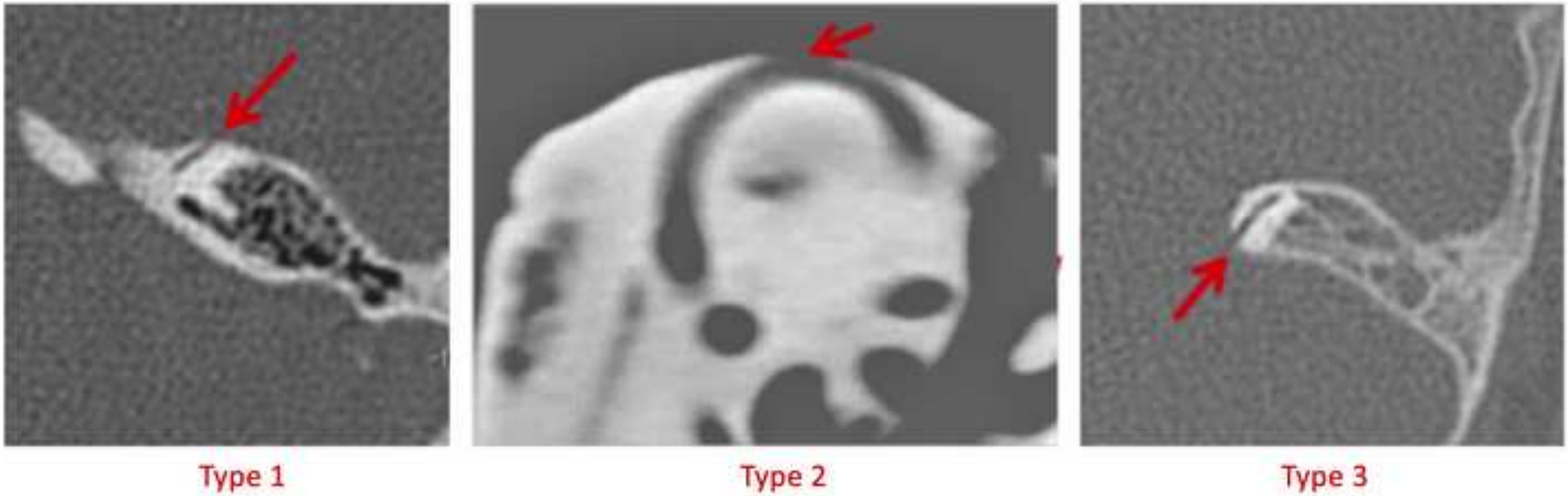

Supplement: Attachment — Submitted filename: PONE-D-21-25618 reviewed.pdf [file pone.0262758.s001.pdf]
