# Supplementary material for: New classification of superior semicircular canal dehiscence in HRCT
Source: PLoS One. 2022 Jan 20;17(1):e0262758. doi: 10.1371/journal.pone.0262758 (PMC8775191; doi:10.1371/journal.pone.0262758)
Supplement: Attachment — Submitted filename: Review of the manuscript PONE-D-21-25618.docx [file pone.0262758.s002.docx]

Review of the manuscript.

PONE-D-21-25618

The review points are added to article text in a yellow sign, that you may find in the attach

The article is an original research

It is a retrospective study

The title indicates well the study design

The abstract is informative but need some more conclusions

The results are well presented but a table in the text should be reviewed

The authors describe limitations

The conclusion are warranted by the results

In my opinion it is an article about an interesting subject in which CT scan findings contribute to diagnosis of superior semicicular dehiscence syndrome.

The article can be accepted to publication after minor review points from the authors which are pointed in the text.

Some of the points:

Frequency of diagnosis and location of SSCD in HRCT and correlation with otoneurological examination. Stablished a SSCD classification BUT IN ORDER TO WHAT FOR?

Should specify what is ENT

The classification is a new classification? In introduction section please mention the reference in the literature or mention that is a new classification that the authors purpose.

Describe better what is considered type 1, 2 or 3, for where to where in superior semicicular canal in imaging.
